# Postoperative Cardiac Arrest after Heart Surgery: Does Extracorporeal Perfusion Support a Paradigm Change in Management?

**DOI:** 10.1155/2010/937215

**Published:** 2010-06-03

**Authors:** Edward Gologorsky, Francisco Igor B. Macedo, Enisa M. Carvalho, Angela Gologorsky, Marco Ricci, Tomas A. Salerno

**Affiliations:** ^1^CVT Division, Department of Anesthesiology, Jackson Memorial Hospital, University of Miami Miller School of Medicine, Miami, FL 33139, USA; ^2^Division of Cardiothoracic Surgery, Jackson Memorial Hospital, University of Miami Miller School of Medicine, Miami, FL 33139, USA; ^3^Department of Anesthesiology, Memorial Regional Hospital, Hollywood, FL 33021, USA

## Abstract

Early institution of extracorporeal perfusion support (ECPS) may improve survival after cardiac arrest. Two patients sustained unexpected cardiac arrest in the Intensive Care Unit (ICU) following cardiac interventions. ECPS was initiated due to failure to restore hemodynamics after prolonged (over 60 minutes) advanced cardiac life support (ACLS) protocol-guided cardiopulmonary resuscitation. Despite relatively late institution of ECPS, both patients survived with preserved neurological function. This communication focuses on the utility of ECPS in the ICU as a part of resuscitative efforts.

## 1. Introduction

The incidence of perioperative cardiac arrest after open heart surgery ranges from 0.7% to 2.9% [1–3] and has decreased in recent years. Survival to hospital discharge of these patients, while higher if compared to other hospital settings, is variable, ranging from 17% to 79% [4]. Extracorporeal perfusion support (ECPS), aimed at improving survival during cardiopulmonary resuscitation (CPR), was introduced by Mattox and Beall over three decades ago [5]. To be effective, ECPS should be initiated early, since outcome of CPR lasting over 10 minutes is dismal. We report two patients who were discharged from hospital with good neurological outcomes, despite rather late initiation of ECPS. All patients signed informed consent, which gives permission for publication of their cases.

## 2. Patient 1

A 69-year-old male with acute non-ST elevation myocardial infarction, underwent off-pump coronary artery bypass grafting, with left internal mammary artery to the left anterior descending coronary artery, and vein grafts to the obtuse marginal and right coronary arteries. Intraoperatively, Doppler flows in all grafts were excellent before and after protamine administration. On postoperative day 2, the patient suddenly developed an episode of ventricular fibrillation, followed by cardiac arrest. Resuscitative efforts were instituted immediately and included closed chest massage, multiple defibrillation attempts, and inotropic support. After twenty minutes of futile closed-chest massage, the sternotomy was reopened, and open cardiac massage was initiated and continued en-route to the operating room (close to thirty-five minutes of open-chest massage). After heparinization, the right atrium and the ascending aorta were cannulated, and cardiopulmonary bypass (CPB) was initiated. The graft to the obtuse marginal artery was found to be occluded. This graft was redone, and good diastolic flow was documented by Doppler flow analysis. Attempts at weaning from CPB were unsuccessful despite inotropic support and intra-aortic balloon pump counter-pulsation. Extra-corporeal membrane oxygenation (ECMO) was initiated using the pre-existing cannulae, with venous return from the right atrium and arterial inflow into the ascending aorta. The sternotomy was left open, and the patient was transported to the ICU, where he was successfully weaned from ECMO over the following twenty-four hours. Three days later, as cardiac function steadily improved and tissue edema subsided, the sternotomy was closed at the bedside. Ensuing multiple organ system failure (renal, respiratory, and sepsis) was treated with dialysis, mechanical ventilatory support, and appropriate antibiotics. Recurrent episodes of VF and low ejection fraction (20%) required subsequent placement of an automatic implantable defibrillator device. The patient was discharged home on postoperative day 32 and has remained well on follow-up.

## 3. Patient 2

A 57-year old male with past history of diabetes, hypertension, and coronary artery disease presented with ventricular tachycardia and fibrillatory arrest (VF). Following resuscitation by paramedics, he continued to have recurrent nonsustained episodes of VF. Emergency cardiac catheterization demonstrated critical left anterior descending artery lesion and greater than 50% left main coronary artery stenosis. Because of recurrent VF, an intra-aortic balloon pump was placed and salvage angioplasty was attempted. Three hours after the procedure, the patient suffered an episode of VF cardiac arrest, unresponsive to closed-chest massage and multiple defibrillation attempts. Closed-chest cardiac massage was continued en-route to the operating room for emergency salvage coronary revascularization (total closed-chest massage time was seventy minutes). After sternotomy, and while closed-chest massage was being performed, the patient was heparinized and CPB was initiated using right atrial outflow and aortic inflow cannulae. Coronary artery revascularization of the left anterior descending and obtuse marginal coronary arteries was performed using saphenous veins. Doppler examination confirmed good diastolic flows in both grafts. As the patient could not be weaned from CPB despite escalating inotropic support, ECMO was initiated using pre-existing cannulae. The sternotomy was left open, and the patient was transported to the ICU. Despite ensuing severe coagulopathy, cardiac function steadily improved over the next forty-eight hours, allowing for successful weaning from ECMO. The sternotomy was closed two days later at the bedside. The postoperative course was complicated by local and systemic infections, requiring six-week course of broad-spectrum antibiotics. He subsequently had an automatic implantable defibrillator device placed and was discharged home in satisfactory condition.

## 4. Discussion

Acute ischemia (such as from a failed coronary artery bypass graft) and delayed postoperative ischemia-reperfusion injury are the major causes of early postoperative cardiac arrest, followed by cardiac tamponade and electrolyte imbalance. Management of these patients is guided by ACLS protocols [6], concomitantly with treatment of the etiological cause [7, 8]. Open cardiac massage may be indicated, if initial resuscitative measures are unsuccessful [9–11]. 

The unsatisfactory results of the ACLS-driven protocols lead some to consider the extracorporeal perfusion as part of resuscitative paradigm [5]. The concept of extracorporeal perfusion support (ECPS) has evolved from cardiopulmonary bypass (CPB) to ECMO and further to extracorporeal cardiopulmonary resuscitation (ECPR) [12, 13]. The goals of the ECPS dictate the choice of the application and the technique. While CPB is designed to isolate the quiescent and nonperfused heart and lungs for the duration of the cardiac procedure, ECMO is aimed at supporting perfused but failing heart and lungs. Examples include acute respiratory distress syndrome, acute myocardial infarction with severe cardiac compromise, and severe postcardiotomy syndrome with failure to separate from CPB. This utilitarian distinction leads to significant differences in the technical aspects. Limited duration of CPB allows for the use of microporous membrane oxygenators, large blood-air and blood-tubing interface areas, and requires higher level of heparinization in order to maintain activated clotting times above 400 seconds. Conversely, the longer duration of ECMO requires smaller priming volumes of hollow fiber oxygenators and leads to smaller blood-air and blood-tubing interface areas, less heparin usage, and reduced coagulopathy. The conceptual integration of extracorporeal perfusion support into the resuscitation led to the development of ECMO-assisted CPR (ECPR). The goal of ECPR is to utilize ECPS to maintain cardiac and pulmonary functions, in the face of imminent death. This approach allows the use of hypothermia and readily achieves important resuscitative goals, such as decreased blood viscosity and washing out of calcium and reactive free radicals, associated with exacerbation of ischemia-reperfusion injury [12–14]. 

 Early institution of ECPS may improve in-hospital survival in pediatric and adult patients [15–18]. AHA guidelines for CPR include consideration for ECMO support as a Class IIb indication, if no-flow period is brief and the cause is reversible [19]. The largest to-date single-center series of patients who had ECMO therapy after cardiac arrest and prolonged (>10 minutes) CPR indicate survival-to-discharge rate of close to 30%, with acceptable neurological status [20]. These trends led to the development of ultraportable preprimed ECMO devices, on stand-by, for prompt flow restoration within minutes after cardiac arrest [21–24]. 

In contrast, the patients herein presented had undergone lengthy periods (close to an hour) of unsuccessful CPR. This is traditionally associated with dismal neurologic outcome. Despite this, with assistance of ECPS, these patients survived with normal neurological function. We would like to suggest two important variables which may account for high survival and hospital discharge rates (75% and 63%, resp.) in patients who suffered cardiac arrest in the ICU: cardiac surgical ICU setting allows for ready sternal re-entry [24], and the etiologic causes of cardiac arrest are often readily identified and reversed. Furthermore, even late initiation of EPS may have mitigated ischemia-reperfusion injury through anticoagulation, hypocalcemia, dilution of free radicals, and achievement of higher perfusion pressure, needed to minimize “no-reflow” injury [25]. On the other hand, ECPS is associated with intense inflammatory response, which exacerbates ischemic and ischemia-reperfusion injury, and may lead to the development of vasoplegia, coagulopathy, infections, neurologic, pulmonary, and vaso-occlusive complications, contributing to both early and late morbidity and mortality. Low hospital ECMO survival rates (16%–41%) reflect both the gravity of the underlying pathology and the complications related to ECP [26–28] but offer hope in face of imminent patient′s demise.

In conclusion, in contrast to the general hospital population, patients after cardiac procedures in the ICU represent a different and largely unexplored cohort. Further investigation is required to address this issue in patients who have sustained cardiac arrest in that setting. In this population of patients, prolonged (60 minutes) CPR does not appear to preclude subsequent ECMO support, allowing for recovery of cardiac and neurological functions.
